# Comparative Study of Bioactive Lipid Extraction from Squid (*Doryteuthis gahi*) by-Products by Green Solvents

**DOI:** 10.3390/foods11152188

**Published:** 2022-07-23

**Authors:** Santiago P. Aubourg, Marcos Trigo, María Jesús González, Salomé Lois, Isabel Medina

**Affiliations:** Department of Food Technology, Marine Research Institute, CSIC, C/E. Cabello, 6, 36208 Vigo, Spain; mtrigo@iim.csic.es (M.T.); mjgp@iim.csic.es (M.J.G.); salomel@iim.csic.es (S.L.); medina@iim.csic.es (I.M.)

**Keywords:** Patagonian squid, by-products, bioactive lipids, green extraction, ethanol, acetone, ethyl acetate, phospholipids, PUFA, ω3/ω6 ratio

## Abstract

A novel approach of bioactive lipid extraction by different green solvents was carried out on squid (*Doryteuthis gahi*) by-products. By-products (viscera, heads, skin, tails, etc.), considered as a single product, were subjected to the following solvent systems: ethanol, acetone, ethyl acetate, 1/1 ethanol/acetone, 1/1 ethanol/ethyl acetate, and 1/1 acetone/ethyl acetate. Analyses carried out included lipid yield, lipid class content, and fatty acid (FA) composition. Results were compared to the lipid extract obtained by the traditional procedure (1/1 chloroform/methanol). Lipid yields obtained by green solvents led to a 33.4–73.2% recovery compared to traditional extraction; the highest values (*p* < 0.05) were obtained by ethanol-containing systems. Compared to the traditional procedure, ethanol systems showed an 85.8–90.3% recovery of phospholipid compounds and no differences (*p* > 0.05) in the ω3/ω6 ratio. Green-extracting systems led to higher average values for eicosapentaenoic acid content (15.66–18.56 g·100 g^−1^ total FAs) and polyene index (1.93–3.29) than chloroform/methanol extraction; differences were significant (*p* < 0.05) for systems including acetone and ethyl acetate. No differences (*p* > 0.05) were detected for docosahexaenoic acid content between the traditional procedure and green systems, with all values being included in the 31.12–32.61 g·100 g^−1^ total FA range. The suitability of EtOH-containing green systems for extraction of bioactive lipid compounds from squid by-products was concluded.

## 1. Introduction

Chemical diversity observed in marine species makes them an extraordinary source of highly valuable constituents such as unsaturated fatty acids, essential minerals, lipid-soluble vitamins, and nutritional and digestible proteins [1]. Marine lipids differ from lipids from other plant and animal sources in that they contain a wider range of fatty acids (FAs), longer-chain FAs, and a larger proportion of highly unsaturated FAs, particularly ω3 FAs such as docosahexaenoic (DHA) and eicosapentaenoic (EPA) acids [2]. Interestingly, the positive role of ω3 FA compounds in preventing certain human diseases (i.e., cardiovascular, neurodegenerative, etc.) has provoked a great deal of attention on marine lipids [3,4].

Fishing activities are reported to generate a high amount of waste. Marine by-products (blood, viscera, heads, bellies, bones, skin, etc.) are a main concern for current fishery management policies and legislations [5]. Remarkably, marine by-products constitute a relevant source of main constituents such as lipids, proteins, minerals, and vitamins, in addition to minor components such as enzymes, amino acids, pigments, chitin, collagen, and other bioactive compounds [6,7]. Consequently, employment of such compounds has been found useful for the development of food, pharmaceutical, nutraceutical, and cosmeceutical industries [8,9].

Concerning the lipid fraction of seafood by-products, great efforts have been carried out on the extraction of valuable constituents with highly nutritional and functional properties for human health such as flavonoids, vitamins A and D, ω3 FA concentrates, phospholipids or polyphenols in general [10,11,12]. Among them, physical fractionation [13], wet pressing [14,15], supercritical fluid extraction [16], urea concentration [17], and enzymatic hydrolysis [18,19] have been tested satisfactorily. Concerning the procedure of solvent extraction, great attention has been paid in recent decades to replace organic solvents with eco-friendly solvents. Remarkably, such efforts have been especially focused on agricultural by-products and have employed different kinds of green solvents such as ethanol, acetone, glycerol, and ethyl acetate [20,21,22]. Concerning green solvent employment on marine products, previous research can be considered scarce. Thus, acetone/ethanol mixtures were tested for lipid class extraction from Antarctic krill (*Euphausia superba*) [23] and ethanol/hexane was checked as an extracting system for obtaining antioxidants (i.e., vitamin E) from microalga (*Scenedesmus dimorphus*) [24].

The present study focused on a novel and alternative extraction of bioactive marine lipids by green solvents. As a marine substrate, by-products obtained after commercialisation of Patagonian squid (*Doryteuthis gahi*) were employed. This species was chosen as being an important fishery resource widely distributed along the Pacific and Atlantic coasts of South America [25]. By-products (viscera, tails, heads, tentacles, skin, etc.), considered as a single product, were lyophilised and then subjected to the following solvent systems: ethanol (EtOH), acetone (AcMe), ethyl acetate (AcOEt), 1/1 EtOH/AcMe (*v/v*), 1/1 EtOH/AcOEt (*v/v*), and 1/1 AcMe/AcOEt (*v/v*). Analyses carried out included lipid yield, lipid class content (phospholipids, PLs; free fatty acids, FFAs; sterols, STs; triacylglycerols, TAGs) and FA content (saturated FAs, STFAs; monounsaturated FAs, MUFAs; polyunsaturated FAs, PUFAs; ω3/ω6 ratio; polyene index, PI). Results were compared to those corresponding to the lipid extract obtained by the traditional procedure (i.e., 1/1 CHCl_3_/MeOH, *v/v*) [26].

## 2. Materials and Methods

### 2.1. Solvents, Chemicals, and Standars

Solvents and chemical reagents used were of reagent grade and purchased from Merck (Darmstadt, Germany). The following solvents were employed: ethanol, acetone, ethyl acetate, chloroform, methanol, hexane, and toluene. The following reagents were used: ammonium molybdate, cupric acetate, pyridine, acetic acid, acetic anhydride, acetyl chloride, NaCl, anhydride sodium sulphate, sulphuric acid, ferric trichloride, perchloric acid, nitric acid, and hydroxylamine.

Quantitative standards (1,2-dipalmitoyl-rac-glycero-3-phosphocholine, oleic acid, cholesterol, methyl stearate, nonadecanoic acid) were purchased from Sigma-Aldrich (St. Louis, MO, USA). Qualitative (Qualmix Fish) and quantitative (FAME Mix) FAME standards were obtained from Supelco, Inc. (Bellefonte, PA, USA) and Larodan (Malmo, Sweden), respectively.

### 2.2. Initial Squid, Lyophilisation, and Extracting Systems

Patagonian squid (*Doryteuthis gahi*) by-products (viscera, heads, skin, tails, etc.) were provided by SERPESBA S. L. U. (Vigo, Spain). The squid were obtained near the Argentinean coast from the southwest Atlantic Ocean. Squid samples were frozen (−40 °C) and transported to the factory in Vigo (Spain) where samples were thawed (overnight storage at 4 °C), the mantel being taken for commercialisation and the resulting by-products pooled together and transported (for 15 min) in refrigerated conditions (4 °C) to our laboratory placed in the same town.

In order to analyse the initial by-products, three × 10 g portions of by-products were separated and subjected to moisture assessment and lipid extraction by the traditional procedure according to the methodology described later on. Lipid extracts were stored at −40 °C before subsequent lipid composition analysis.

On the same day, two × 1 kg of by-products were subjected to the lyophilisation process (−70 °C, 72 h, 0.05 mTorr) (Model FD8515-C60, Ilshin Biobase Europe, Ede, The Netherlands). After this time, lyophilised samples were pooled together, minced, and employed for lipid extraction by different extracting mixtures. For it, the following green solvent systems were applied: EtOH, AcMe, AcOEt, 1/1 EtOH/AcMe (*v/v*), 1/1 EtOH/AcOEt (*v/v*), and 1/1 AcMe/AcOEt (*v/v*). Additionally, lipid extraction by the traditional procedure (1/1 CHCl_3_/MeOH, *v/v*) was also carried out.

According to preliminary trials carried out in our laboratory, the lyophilisation step was found necessary in order to substantially increase the lipid and PL yields by using the green solvent mixtures. This procedure, including moisture elimination in general, has already been described as necessary in previous related research including green solvents such as acetone and ethanol [23,27].

### 2.3. Moisture Assessment, Lipid Extraction, and Lipid Yield

Moisture was determined in initial by-products and in lyophilised by-products as the weight difference (1–2 g) before and after 4 h at 105 °C according to official method 950.46 B [28]. Results were calculated as g·kg^−1^ squid by-products.

Traditional lipid extraction of initial by-products and lyophilised by-products was carried out by the Bligh and Dyer [26] method; this procedure employs a single-phase solubilisation of the lipids using a CHCl_3_/MeOH (1/1) mixture.

Lipid extraction of lyophilised by-products by employment of the above-mentioned green solvent systems was carried out as follows: 3.5 g of lyophilised by-products were mixed with 10 mL of the extracting system, stirred for 1 min at 4 °C, centrifuged at 3500× *g* for 10 min at 4 °C, and the supernatant was collected. The procedure was repeated two more times, with all supernatants being collected together. The resulting extracts were subjected to partial evaporation of the solvent (rotary evaporator; 10 min at 30 °C), and were brought up to a 15 mL volume and stored at −40 °C before analysis of lipid composition.

Each of the extracting systems (traditional and green) was carried out in triplicate (*n* = 3). In all kinds of lipid extracts, quantification of lipid extracts was carried out according to Herbes and Allen [29]. Results were calculated as g·kg^−1^ by-products.

### 2.4. Lipid Class Analysis

The PL content of lipid extracts was spectrophotometrically (710 nm; Beckman Coulter DU 640 spectrophotometer, Brea, CA, USA) measured according to the method of Raheja et al. [30], which is based on the formation of a coloured complex with ammonium molybdate. For quantification purposes, different quantities (0, 5, 10, 20, 40, 60, 80, 100, 130, and 150 μL) of a 1,2-dipalmitoyl-rac-glycero-3-phosphocholine (DPPC) solution in chloroform (15.3 mg/5 mL) were employed. The validity range was 16.1–483.0 μg DPPC and the R^2^ value of the analytical procedure was 0.9995. Results were calculated as g DPPC·kg^−1^ lipids and g DPPC·kg^−1^ by-products.

The FFA content of lipid extracts was spectrophotometrically (715 nm) determined following the method of Lowry and Tinsley [31], which is based on the formation of a complex with cupric acetate-pyridine. For quantification purposes, different quantities (0, 5, 10, 20, 40, 60, 80, 100, 130, and 150 μL) of an oleic acid solution in toluene (705.3 mg/25 mL) were employed. The validity range was 0.5–15.0 μmol oleic acid and the R^2^ value of the analytical procedure was 0.9998. Results were calculated as g oleic acid·kg^−1^ lipids and g oleic acid·kg^−1^ by-products.

The ST content in lipid extracts was assessed spectrophotometrically (615 nm) by the method of Huang et al. [32], based on the reaction with acetic anhydride in acetic acid (Liebermann–Buchard reaction). For quantification purposes, different quantities (0, 5, 10, 20, 40, 60, 80, 100, 130, and 150 μL) of a cholesterol solution in acetic acid (12.2 mg/5 mL) were employed. The validity range was 11.5–345.0 μg cholesterol and the R^2^ value of the analytical procedure was 0.9998. Results were calculated as g choleterol·kg^−1^ lipids and g choleterol·kg^−1^ by-products.

To evaluate the TAG presence, lipid extracts were first purified on 20 × 20 cm thin-layer chromatography plates coated with a 0.5 mm-layer of silica gel G from Merck using a mixture of hexane-ethyl ether-acetic acid (90/10/1, *v/v*/*v*; two times) as eluent [33]. Once the TAG fraction was purified, the spectrophotometric (520 nm) method of Vioque and Holman [34] was used to measure the ester linkage content according to the conversion of the esters into hydroxamic acids and subsequent complexion with Fe (III). For quantification purposes, different quantities (0, 2, 5, 10, 20, and 40 μL) of a methyl stearate solution in toluene (41.0 mg/5 mL) were employed. The validity range was 16.4–328.0 μg methyl stearate and the R^2^ value of the analytical procedure was 0.9995. Results were calculated as g tristearine·kg^−1^ lipids and g tristearine·kg^−1^ by-products.

### 2.5. Analysis of the FA Composition 

Lipid extracts were converted into fatty acid methyl esters (FAMEs) by using acetyl chloride in methanol and then analysed by gas–liquid chromatography (GLC; PerkinElmer 8700 chromatograph, Madrid, Spain) [33]. The quantitative response of the equipment was checked with a GLC quantitative standard (FAME Mix, Supelco, Inc., Bellefonte, PA, USA). Peaks corresponding to FAMEs were identified by comparison of their retention times with those of a standard mixture (Qualmix Fish, Larodan, Malmo, Sweden). Peak areas were automatically integrated. C19:0 FA was used as internal standard for quantitative purposes; for it, 100 μL (i.e., 40 μg C19:0) of a 0.4 mg·mL^−1^ solution in toluene were added to each sample before the methylation reaction with acetyl chloride. Limits of detection and quantification were 500 and 1500 area units, respectively. The content of each FA was calculated as g·100 g^−1^ total FAs.

Results concerning FA groups (STFAs, MUFAs, PUFAs, ω3 FAs, and ω6 FAs) and FA ratios (ω3/ω6 and EPA/DHA) were calculated according to quantification of individual FA compounds. Additionally, the PI was calculated on the basis of the following FA content ratio [33]: (EPA + DHA)/C16:0.

### 2.6. Statistical Analysis

Data (*n* = 3) obtained from the different lipid analyses (yield, lipid classes, and FA profile and ratios) were subjected to a one-way ANOVA (*p* < 0.05) to investigate differences among the different kinds of lipid extracts (traditional and green systems) (Statistica version 6.0, 2001; Statsoft Inc., Tulsa, OK, USA). Comparison of means was performed using a least-squares difference (LSD) method.

## 3. Results and Discussion

### 3.1. Determination of Moisture and Lipid Contents 

Initial by-products employed in the current study showed a moisture value of 842.6 ± 2.2 g·kg^−1^. Concerning the lipid content of the initial sample, extraction carried out by the traditional procedure led to a level of 19.0 ± 0.5 g·kg^−1^ by-products (Table 1). Values for both constituents agree with those obtained recently during a seasonal study of by-product composition of the same squid species [35]; at that time, value ranges detected for moisture and lipids were 829.0–842.8 and 17.5–21.8 g·kg^−1^ by-products, respectively. Previous research related to moisture and lipid content of cephalopod by-products shows varying values according to the species and the particular body tissue taken into account. Thus, similar values as found in the current study were obtained by Kacem et al. [36] for moisture (75–85%), and lipids (0.58–4.02%) in viscera fractions (stomach, intestines, and pyloric caeca) from *Sepia officinalis* captured off the Tunisian coasts at different seasons. However, squid (*Loligo formosana*) ovary showed lower levels for both constituents, with values obtained being 72.1% and 0.5% for moisture and lipids, respectively [37]. Contrary to these results, a higher lipid content (199.8 g·kg^−1^ viscera) than in the present study was detected by Toyes-Vargas et al. [38] in giant squid (*Dosidicus gigas*) viscera. Notably, a marked difference in lipid content was detected by Saito et al. [39] when analysing the liver (15.7–17.9%) and gonad (1.0–1.4%) of Humboldt squid (*Dosidicus gigas*).

The current lyophilisation process of squid by-products led to a substantial moisture loss, so that a 52.1 ± 0.9 g·kg^−1^ value was detected. Therefore, lipid extraction carried out by all extracting systems tested led to a substantial content increase in this constituent (Table 1) when compared to initial by-products; remarkably, the highest values (*p* < 0.05) were obtained by applying the traditional procedure. Concerning the green solvent extraction, values detected were included in the 36.3–73.6 g·kg^−1^ by-products range (Table 1); this corresponded to a 33.4–73.2% recovery when compared to the lipid yield obtained in lyophilised samples by the traditional procedure. This different recovery can be explained on the basis that green solvents tested are more polar than the chloroform/methanol mixture and, therefore, would not be likely to extract entirely non-polar lipid classes such as TAGs, waxes, cholesterol esters, etc. On the other side, great differences were found among the different extracting systems tested. Thus, those including EtOH led to higher (*p* < 0.05) lipid recoveries than in the case of employing extracting systems not including this solvent. Remarkably, the EtOH/AcMe-extracting system reached the highest average recovery (i.e., 73.2%). In previous research, Gigliotti et al. [23] tested the lipid extraction from Antarctic krill (*Euphausia superba*) with different AcMe/EtOH ratios; as in the present research, an increasing proportion of ethanol led to a lipid yield increase so that higher lipid levels were obtained by using solvent ratios (*v/v*) of 1/30 (ca. 13.5%) and 1/12 (ca. 12.0%) than with 1/9 (ca. 9.5%) and 1/6 (ca. 7.5%) ratios.

### 3.2. Determination of Lipid Classes

**Figure 1:**
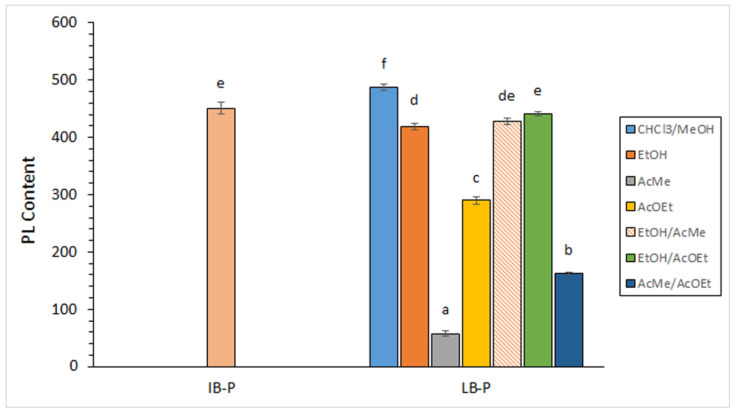
Determination of phospholipid (PL) content (g·kg^−1^ lipids) in initial by-products (IB-P) and lyophilised by-products (LB-P) obtained by different lipid-extracting systems. Average values of three independent determinations (*n* = 3); standard deviations are indicated by bars. Values accompanied by different lowercase letters (a–f) indicate significant differences (*p* < 0.05). Extracting systems as expressed in Table 1.

Initial by-products showed PL values included in the 450.8 ± 9.9 g·kg^−1^ lipid range (0.86 ± 0.03 g·kg^−1^ by-products) (Figure 1), according to a previous report on by-products from the same squid species measured during a seasonal study (359.2–463.5 g·kg^−1^ lipids) [34]. This PL presence in the lipid fraction can be considered similar to the one reported in edible parts of lean fish species such as rainbow trout (*Oncorhynchus mykiss*) [40] and megrim (*Lepidorhombus whiffiagonis*) [41].

Comparison between extracts obtained by the traditional procedure on initial by-products and lyophilised by-products revealed a slight increase (*p* < 0.05) as a result of the lyophilisation process (Figure 1). Concerning green solvent extraction, varying PL values were detected, all of them lower than that obtained by the traditional procedure (Figure 1). Among green systems, the highest levels (*p* < 0.05) were obtained by applying systems including EtOH (418.5–440.4 g·kg^−1^ lipids; 2.75–3.15 g·kg^−1^ by-products). On the contrary, the lowest levels (*p* < 0.05) were detected for the AcMe solvent (57.6 g·kg^−1^ lipids; 0.21 g·kg^−1^ by-products). It is worth pointing out that systems including EtOH provided recovery values accounting for 85.8–90.3% when compared to CHCl_3_/MeOH extraction. Therefore, EtOH-containing systems can be considered an interesting green procedure in order to obtain this highly valuable lipid class. A greater recovery with EtOH-containing systems can be explained on the basis of the higher polarity of EtOH than that of AcMe and AcOEt, so that polar lipids such as PL classes would be extracted more abundantly. Contrary to these extractability results, a higher PL yield (ca. 30%) was detected by applying a 1/6 ratio (AcMe/EtOH) than in the case of using a higher presence of ethanol (1/9, 1/12, and 1/30, AcMe/EtOH; ca. 21–22%) during PL extraction from krill (*E. superba*) [23].

PL compounds have been described as being important constituents of cell membranes and having an important structural role in living bodies in general. Furthermore, and on the basis of their amphiphilic character, PLs have recently attracted great attention for serving as drug delivery systems and having a high bioavailability and protecting effect on different kinds of diseases [42,43]. Thus, profitable functions related to pharmaceutical and food production industries have recently been developed for marine PL compounds [12]. Therefore, the present study has shown that EtOH-including systems can provide an accurate way of extracting valuable PL compounds by a green procedure.

Results obtained for FFA and ST classes are presented in Table 1. Average values obtained in initial by-products for both lipid classes (228.2 g FFA·kg^−1^ lipids and 4.34 g FFA·kg^−1^ by-products; 103.6 g ST·kg^−1^ lipids and 0.20 g ST·kg^−1^ by-products), agree with the previous research obtained during a seasonal study on by-products corresponding to the same squid species [35]; in that study, values obtained for FFA and ST classes were included in the ranges 156.6–282.0 and 115.0–132.1 g·kg^−1^ lipids, respectively. Compared to values detected in edible parts of cephalopod species and marine species in general [33,44,45], FFA levels of current by-products can be considered relatively high. Such high values can be explained on the basis of the great presence of lipases and phospholipases in visceral tissues [7,19]. Thus, higher FFA levels (g·100 g^−1^ tissue) were detected in ovary (0.03–0.10) and hepatopancreas (0.18–0.59) than in arm (0.01) and mantle (0.01) during a seasonal study on common octopus (*O. vulgaris*) [46]; when compared to present by-products, ovary FFA values were found to be lower, but FFA presence in hepatopancreas was found to be similar. Higher FFA levels than in the present study were detected in *Loligo duvauceli* liver (10.67 g·kg^−1^ tissue) by Vairamani et al. [47]. Concerning previous research on the ST level, higher values were also obtained in ovary (0.16–0.31 g·100 g^−1^ tissue) and hepatopancreas (0.28–0.69 g·100 g^−1^ tissue) than in edible tissues such as arm (0.03–0.05 g·100 g^−1^ tissue) and mantle (0.03–0.06 g·100 g^−1^ tissue) during a seasonal study on octopus (*O. vulgaris*) [46]; a comparison to present by-products showed that hepatopancreas values were found to be higher, but ovary presence was found to be similar. 

Comparison between lipid extracts obtained by the traditional procedure on initial by-products and lyophilised by-products revealed an average decrease in FFA and ST contents as a result of lyophilisation (Table 1); this decrease was found significant (*p* < 0.05) in the case of the FFA value. Compared to the traditional extraction on lyophilised by-products, all green-extracting systems led to an increased average value of both lipid classes. Among the green-extracting systems, the following increasing (*p* < 0.05) sequence for solvent extractability was observed: EtOH < AcOEt < AcMe; remarkably, the highest (*p* < 0.05) proportions of both lipid classes were detected in the extract corresponding to the AcMe system. Results obtained for the comparative extractability of the three green solvents can be explained on the basis of their relative polarities; thus, a lower polarity of AcMe than that of EtOH and AcOEt has shown a higher ability for extracting lipid classes such as FFAs and STs. According to this result, Gigliotti et al. [23] observed an increasing cholesterol yield by increasing the AcMe presence in an AcMe/EtOH-extracting system when applied to Antarctic krill (*E. superba*).

A very low content of TAG compounds was detected in initial by-products (Table 1). Such samples showed slightly lower values than those reported for by-products from the same squid species (9.5–13.1 g·kg^−1^ lipids) [35]. Additionally, present TAG levels (0.02 ± 0.00 g·kg^−1^ by-products) were found lower than those detected by Sieiro et al. [46] in a seasonal study carried out on the ovary (0.32–0.52 g·100 g^−1^ tissue) and the digestive gland (2.51–3.71 g·100 g^−1^ tissue) of octopus (*O. vulgaris*).

This low TAG presence in the lipid fraction was maintained in the lipid extract of lyophilised by-products obtained by the traditional procedure (Table 1). Remarkably, average values obtained by all kinds of green extraction solvents were found lower than those obtained by the traditional procedure; differences were found significant (*p* < 0.05) in all cases except for the comparison to the AcMe system (*p* > 0.05). Comparison among extracting systems led to a similar trend in the case of the FFA and ST classes. Thus, extracting systems including EtOH led to lower (*p* < 0.05) TAG levels than systems not including this solvent. Furthermore, the AcMe solvent provided the highest average levels of this lipid class. As for FFA and ST classes, TAG results obtained concerning the comparative ability of the extracting solvents tested can be explained on the basis of their different polarities. As being a non-polar lipid class, TAGs have been extracted more extensively by the less polar solvent tested (i.e., AcMe). Contrary to the present results, Gigliotti et al. [23] showed that TAG extraction in lipids from Antarctic krill (*E. superba*) increased with the ethanol presence in the AcMe/EtOH-extracting system; thus, the 1/12 ratio showed higher TAG yields (ca. 2%) than 1/6 and 1/9 (ca. 1%) ratios.

### 3.3. Fatty Acid (FA) Analysis

**Figure 2:**
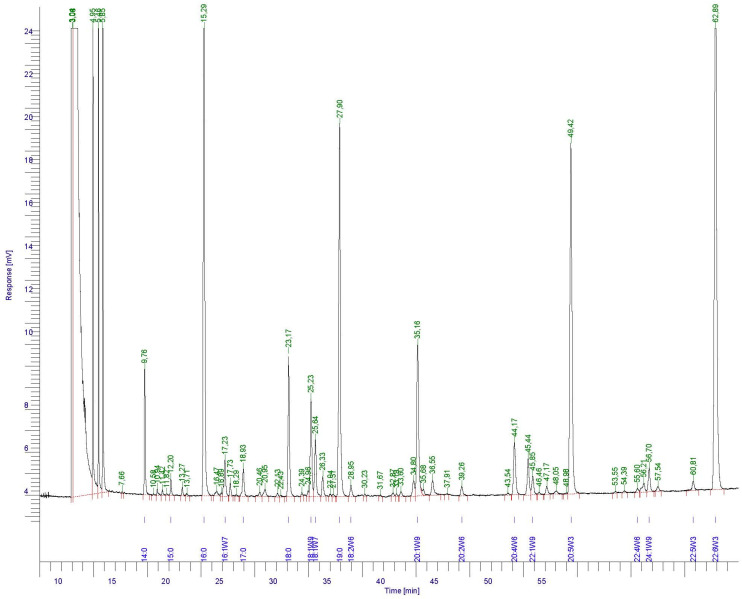
Fatty acid (FA) profile of initial by-products. Retention time (min) of individual FA is indicated in green. Assignation of peaks is expressed in blue.

The FA profile of initial by-products is shown in Figure 2. Composition (g·100 g^−1^ total FAs) was as follows: 3.19 ± 0.12 (C14:0), 0.50 ± 0.03 (C15:0), 25.45 ± 0.87 (C16:0), 1.51 ± 0.10 (C16:1ω7), 1.18 ± 0.04 (C17:0), 4.88 ± 0.05 (C18:0), 3.68 ± 0.05 (C18:1ω9), 2.25 ± 0.06 (C18:1ω7), 0.50 ± 0.01 (C18:2ω6), 5.66 ± 0.05 (C20:1ω9), 0.48 ± 0.04 (C20:2ω6), 2.53 ± 0.09 (C20:4ω6), 0.78 ± 0.04 (C22:1ω9), 15.88 ± 0.04 (C20:5ω3, EPA), 0.21 ± 0.02 (C22:4ω6), 0.89 ± 0.03 (C24:1ω9), 0.48 ± 0.01 (C22:5ω3), and 29.95 ± 0.01 (C22:6ω3, DHA).

According to such profile, DHA, C16:0, and EPA showed to be the most abundant FA compounds present in the squid by-products. Other relatively abundant FA components were C20:1ω9, C18:0, C18:1ω9, C14:0, and C20:4ω6. This FA composition agrees with previous results obtained on by-products corresponding to the same squid species [35]. A similar distribution of the main FA constituents (i.e., DHA, EPA, C16:0, C18:0, C20:4ω6, C18:1ω9, and 20:1ω9) was detected for ovary and digestive gland from octopus (*O. vulgaris*) [46].

Among the ω3 PUFAs, great attention has been accorded to DHA and EPA values in view of their beneficial health effects. Thus, clinical and epidemiological studies have associated EPA consumption with low prevalence of coronary, circulatory, and inflammatory diseases [48], whereas DHA has been related to foetal development, prevention of neurodegenerative diseases, and correct functioning of the nervous system and visual organs in the foetus [49]. In the present study, a comparison between traditional lipid extracts of initial by-products and lyophilised by-products showed that the lyophilisation process led to an average value increase for both PUFA compounds; such increase was found significant (*p* < 0.05) for DHA (Table 2). Increased average DHA values were also detected in all green-extracting systems when compared to the traditional procedure, although differences were not found significant (*p* > 0.05). In the case of EPA, lower average values were detected in samples corresponding to EtOH-extracting systems when compared to the traditional procedure; on the contrary, higher levels (*p* < 0.05) were obtained in AcMe-, AcOEt-, and AcMe/AcOEt-extracting systems when compared to the CHCl_3_/MeOH system.

Previous research on cephalopod by-products has shown varying values for DHA and EPA content, according to the species and the body tissue taken into account. Thus, Kacem et al. [36] found lower values than in the current study for DHA (9.1 g·100 g^−1^ total FAs) and EPA (11.6 g·100 g^−1^ total FAs) during a seasonal study in cuttlefish (*S. officinalis*) viscera. Toyes-Vargas et al. [38] denoted levels of 19.4 and 16.9 g·kg^−1^ viscera in giant squid (*D. gigas*) viscera for DHA and EPA, respectively, which can be considered similar for EPA but lower for DHA when compared to the present values. Recently, Šimat et al. [50] analysed the presence of both PUFA constituents in different kinds of fish by-products; thus, the lowest values (g·100^−1^ g total FAs) were detected in EPA (ca. 3.3) and DHA (ca. 8.3) for sea bass (*Sparus aurata*) and seabream (*Dicentrarchus labrax*) by-products, whereas the highest levels were found in by-products from tuna (*Thunnus thynnus*) and sardine (*Sardina pilchardus*) (9–14 and 13–21, for EPA and DHA, respectively). Therefore, present values obtained for DHA and EPA can be considered as relatively high for a by-product and be valuable as a source of both essential FA compounds.

Comparison among FA group contents showed the following decreasing (*p* < 0.05) tendency in initial by-products (Table 2): PUFA > STFA > MUFA; notably, PUFA presence reached ca. 50% value. Such marked differences among FA groups agree with previous related research on cephalopod by-products [35,46] and were maintained in all kinds of lipid extracts corresponding to lyophilised samples. Comparison of lipid fractions obtained by a traditional extracting procedure on initial by-products and lyophilised by-products showed that the lyophilisation process led to average decreases in STFA and MUFA contents and an average increase in PUFA group value; differences were only found significant (*p* < 0.05) in the case of the MUFA group. Compared to the traditional procedure, green solvent systems provided lower average STFA values and higher average MUFA and PUFA levels; for the MUFA group, such higher values were found significant (*p* < 0.05) for all green systems. 

Comparison among green-extracting systems showed that the highest average values for STFAs were found in samples corresponding to extracting systems including EtOH, whereas the lowest average value was obtained for the AcMe system (Table 2). For the MUFA group, the highest value (*p* < 0.05) was obtained in samples corresponding to AcMe extraction, with the lowest average values obtained in lipid extracts corresponding to the extracting systems including EtOH. Concerning the PUFA group, the highest average value was found in samples corresponding to the AcMe/AcOEt-extracting system; on the contrary, the lowest average values were obtained for systems including EtOH. Remarkably, all green-extracting systems tested led to values included in the 51.8–55.9% range for the PUFA group.

Previous research has paid great attention to the ω3/ω6 ratio on the basis that most Western countries do not consume adequate levels of ω3 FA compounds [51,52]. In order to prevent inflammatory, cardiovascular, and neurological disorders, the World Health Organization (WHO) currently recommends that this ratio should not be below 1/10 in the human diet [53]; additionally, the European Nutritional Society reported that a human diet with an ω3/ω6 ratio of 1/5 or higher would have health benefits [54].

**Figure 3:**
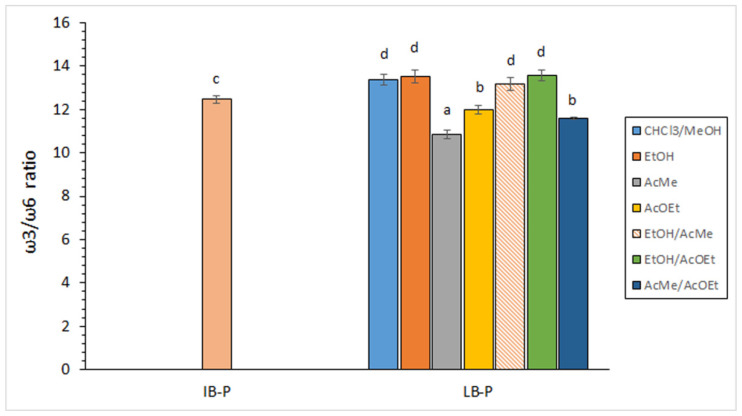
Determination of the ω3/ω6 ratio in initial by-products (IB-P) and lyophilised by-products (LB-P) obtained by different lipid-extracting systems. Average values of three independent determinations (*n* = 3); standard deviations are indicated by bars. Values accompanied by different lowercase letters (a–d) indicate significant differences (*p* < 0.05). Extracting systems as expressed in Table 1.

Values obtained for this FA ratio are presented in Figure 3 and agree with previous research on by-products corresponding to the same squid species [35]. Comparison between lipid fractions obtained by the traditional procedure on initial by-products and lyophilised samples showed a slight increase (*p* < 0.05) of this ratio as a result of the lyophilisation process. Additionally, lower average values were detected in lipid extracts corresponding to systems including AcMe, AcOEt, or both solvents when compared to the traditional procedure. Remarkably, extracting systems including EtOH did not provide differences (*p* > 0.05) with extracts corresponding to the traditional procedure, with all values being included in the 13.18–13.57 range. In spite of differences found for some green extraction systems, and according to the above-mentioned recommended levels for the ω3/ω6 ratio, all values obtained by the green extraction procedures can be considered as highly valuable for the human diet.

Present ω3/ω6 ratio values can be considered similar to those obtained for edible tissues of wild fish species such as blackspot seabream (*P. bogaraveo*) [33] and megrim (*L. whiffiagonis*) [41]. On the contrary, previous studies on seafood by-products have shown lower ω3/ω6 values than in the present research. Thus, Šimat et al. [50] obtained values included in the 6–10 range for tuna (*T. thynnus*) and sardine (*S. pilchardus*) by-products, whereas markedly lower ratios (0–2 range) were obtained for tuna (*T. thynnus*) liver and sea bass (*S. aurata*) and seabream (*D. labrax*) by-products. Lower ω3/ω6 ratio values (5.07 ± 0.02) than in the current study were also obtained by Chakraborty and Joseph [27] when extracting edible tissues from Indian sardine (*S. longiceps*) by applying the cooking and wet-pressing procedure. Recently, Rodríguez et al. [15] obtained ω3/ω6 ratio values included in the 7.6–8.0 range for squid (*Illex argentinus*) viscera.

The assessment of the PI has recently attracted great attention as a way of measuring the possible increase or decrease in the PUFA presence in the lipid fraction during seafood processing in general, and is directly related to the nutritional value of the corresponding lipid food [3,4]. Results obtained in the present study for this index are depicted in Table 2, with all values included in the 1.80–3.29 range. Such values can be considered similar to those obtained in previous research for squid *Illex argentinus* viscera (1.65–2.10 range) [15] and for squid (*D. gahi*) by-products [35].

Comparison between lipid fractions of initial by-products and lyophilised by-products extracted with the traditional procedure showed that the lyophilisation process did not produce changes (*p* > 0.05) in the PI. Furthermore, lipid fraction obtained from by-products by employing extracting systems including EtOH did not reveal differences (*p* > 0.05) with samples extracted with CHCl_3_/MeOH. On the contrary, higher (*p* < 0.05) PI scores were detected in lipid fractions obtained by applying extracting systems including AcMe and AcOEt when compared to the traditional procedure. Remarkably, values obtained for all green-extracting systems were included in the 1.93–3.29 range, which can be considered as highly valuable levels and very similar to values reported for edible tissues obtained from marine species such as megrim (*L. whiffiagonis*) [41], rainbow trout (*O. mykiss*) [40], or blackspot seabream (*P. bogaraveo*) [33].

## 4. Conclusions

A novel approach of bioactive lipid extraction by different green solvent systems was carried out on Patagonian squid (*Doryteuthis gahi*) by-products. As a result, lipid yields obtained by green solvent systems led to a 33.4–73.2% recovery when compared to traditional extraction; remarkably, the highest values (*p* < 0.05) were obtained by EtOH-containing systems. Compared to the traditional procedure, EtOH systems showed an 85.8–90.3% recovery of PL compounds and no differences (*p* > 0.05) in the ω3/ω6 ratio. Green-extracting systems led to higher average values for EPA content (15.66–18.56 g·100 g^−1^ total FAs) and PI (1.93–3.29) than CHCl_3_/MeOH extraction; differences were found significant (*p* < 0.05) for systems including AcMe and AcOEt. No differences (*p* > 0.05) were detected for DHA content between the traditional procedure and green systems, with all values being included in the 31.12–32.61 g·100 g^−1^ total FA range.

The suitability of green solvent systems tested is concluded. Their employment as an alternative method for lipid extraction could be recommended with the aim of reducing the risk of chemical exposure to humans and the environment. Additionally, by-products resulting from Patagonian squid commercialisation are considered in the current work as a single product, implying a simplified handling procedure for subsequent industrial use. Current results revealed that lipid extraction from the present by-products by green processing can be an interesting option for recovering high-value compounds such as PLs, DHA, EPA, and ω3 PUFAs in general, as well as a lipid extract including valuable ω3/ω6 and PI ratios. Remarkably, EtOH-containing systems have shown the highest levels of lipid yield, PL recovery, and ω3/ω6 ratio. On the basis of the high relevance of the present results, further research is envisaged to optimise and scale-up the extraction conditions (i.e., green solvent mixtures) of bioactive lipid compounds for the utilisation of the present squid by-products as a source of valuable marine constituents susceptible to be used in food, pharmaceutical, and nutraceutical industries.

## Figures and Tables

**Table 1:** Determination * of lipid yield and lipid class content of initial by-products and lyophilised by-products obtained by different lipid-extracting systems **.

Substrate	Extracting System	Lipid Determination			
		Lipid Yield (g·kg^−1^ by-products)	FFAs (g·kg^−1^ lipids)	STs (g·kg^−1^ lipids)	TAGs (g·kg^−1^ lipids)
Initial by-products	CHCl_3_/MeOH	19.0 ± 0.5 a	228.2 ± 6.1 c (4.34)	103.6 ± 2.6 a (0.20)	8.5 ± 0.1 c (0.02)
Lyophilised by-products	CHCl_3_/MeOH	103.8 ± 1.8 e	194.4 ± 6.7 a (2.02)	102.6 ± 4.8 a (1.06)	8.4 ± 0.7 c (0.09)
	EtOH	65.6 ± 4.2 d	210.1 ± 4.5 b,c (1.38)	138.5 ± 2.6 b (0.91)	1.2 ± 0.7 a (0.01)
	AcMe	36.3 ± 0.4 b	366.3 ± 14.8 f (1.33)	260.0 ± 9.5 e (0.94)	7.0 ± 0.7 b,c (0.03)
	AcOEt	46.8 ± 1.6 c	256.7 ± 8.0 d (1.20)	188.3 ± 6.9 c (0.88)	6.1 ± 1.1 b (0.03)
	EtOH/AcMe	73.6 ± 2.7 d	197.72 ± 2.2 a (1.46)	127.8 ± 6.7 b (0.94)	1.6 ± 0.3 a (0.01)
	EtOH/AcOEt	71.1 ± 1.2 d	201.3 ± 2.9 a,b (1.43)	132.5 ± 1.1 b (0.94)	2.2 ± 0.9 a (0.02)
	AcMe/AcOEt	41.6 ± 2.1 c	307.6 ± 2.4 e (1.28)	232.0 ± 14.8 d (0.97)	5.4 ± 1.0 b (0.02)

* Results expressed as average values of three independent determinations (*n* = 3) ± standard deviations. Data included in brackets correspond to average contents of lipid classes expressed as g·kg^−1^ by-products. In each column, different lowercase letters (a–f) indicate significant differences (*p* < 0.05). ** Extracting systems: 1/1 chloroform/methanol (CHCl_3_/MeOH), ethanol (EtOH), acetone (AcMe), ethyl acetate (AcOEt), 1/1 ethanol/acetone (EtOH/AcMe), 1/1 ethanol/ethyl acetate (EtOH/AcOEt), and 1/1 acetone/ethyl acetate (AcMe/AcOEt). Other abbreviations employed: FFAs (free fatty acids), STs (sterols), and TAGs (triacylglycerols).

**Table 2:** Fatty acid (FA) analysis (g·100 g^−1^ total FAs) * in initial by-products and lyophilised by-products obtained by different lipid-extracting systems **.

Substrate	Extracting System	FA Determination					
		STFAs	MUFAs	PUFAs	DHA	EPA	PI
Initial by-products	CHCl_3_/MeOH	35.20 ± 1.10 d	14.47 ± 0.06 b	50.03 ± 1.13 a	29.95 ± 0.17 a	15.88 ± 0.04 a,b	1.80 ± 0.10 a
Lyophilised by-products	CHCl_3_/MeOH	35.10 ± 0.94 d	13.73 ± 0.07 a	51.17 ± 0.89 a,b	31.12 ± 0.72 b	16.05 ± 0.16 b	1.83 ± 0.08 a
	EtOH	32.58 ± 0.96 c	15.40 ± 0.48 c	52.02 ± 0.50 b	32.26 ± 0.44 b	15.72 ± 0.08 a	1.98 ± 0.09 a
	AcMe	23.35 ± 0.31 a	21.35 ± 0.41 f	55.30 ± 0.66 d	31.47 ± 0.51 b	18.48 ± 0.25 c	3.29 ± 0.09 b
	AcOEt	26.12 ± 1.18 b	18.03 ± 0.18 d	55.85 ± 1.35 c,d	32.61 ± 1.04 b	18.34 ± 0.27 c	2.82 ± 0.21 b
	EtOH/AcMe	33.22 ± 0.53 c	14.90 ± 0.20 b,c	51.88 ± 0.33 a,b	31.92 ± 0.20 b	15.82 ± 0.16 a,b	1.93 ± 0.04 a
	EtOH/AcOEt	33.04 ± 1.61 c,d	14.59 ± 0.06 b	52.37 ± 1.59 a,b,c	32.58 ± 1.25 b	15.66 ± 0.27 a,b	1.98 ± 0.17 a
	AcMe/AcOEt	24.49 ± 1.54 a,b	19.60 ± 0.23 e	55.91 ± 1.31 c,d	32.28 ± 1.04 b	18.56 ± 0.17 c	3.09 ± 0.28 b

* Results expressed as average values of three independent determinations (*n* = 3) ± standard deviations. In each column, different lowercase letters (a–f) indicate significant differences (*p* < 0.05). ** Abbreviations employed: STFAs (saturated fatty acids), MUFAs (monounsaturated fatty acids), PUFAs (polyunsaturated fatty acids), DHA (docosahexaenoic acid), EPA (eicosapentaenoic acid), and PI (polyene index). Extracting systems as expressed in Table 1.

## Data Availability

All related data and methods are presented in this paper. Additional inquiries should be addressed to the corresponding author.

## References

[1] Tilami S.K., Sampels S. (2018). Nutritional Value of Fish: Lipids, Proteins, Vitamins, and Minerals. Rev. Fish. Sci..

[2] Aubourg S.P., Nollet L., Toldrá F. (2010). Lipid compounds. Handbook of Seafood and Seafood Products Analysis.

[3] Minihane A., Armah C., Miles E., Madden J., Clark A., Caslake M., Calder P. (2016). Consumption of fish oil providing amounts of eicosapentaenoic acid and docosahexaenoic acid that can be obtained from the diet reduces blood pressure in adults with systolic hypertension: A retrospective analysis. J. Nutr..

[4] Schunck W., Konkel A., Fischer R., Weylandt K. (2018). Therapeutic potential of omega-3 fatty acid-derived epoxy eicosanoids in cardiovascular and inflammatory diseases. Pharmacol. Ther..

[5] Ferraro V., Cruz I.B., Jorge R.F., Malcata F.X., Pintado M.E., Castro P.M.L. (2010). Valorisation of natural extracts from marine source focused on marine by-products: A review. Food Res. Int..

[6] Rubio-Rodríguez N., Beltrán S., Jaime I., de Diego S.M., Sanz M.T., Carballido J.R. (2010). Production of omega-3 polyunsaturated fatty acid concentrates: A review. Innov. Food Sci. Emerg. Technol..

[7] Atef M., Ojagh M. (2017). Health benefits and food applications of bioactive compounds from fish byproducts: A review. J. Funct. Foods.

[8] Shahidi F. (2007). Maximising the Value of Marine By-Products.

[9] Vázquez J.A., Meduiña A., Durán A.I., Nogueira M., Fernández-Compás A., Pérez-Martín R.I., Rodríguez-Amado I. (2019). Production of valuable compounds and bioactive metabolites from by-products of fish discards using chemical processing, enzymatic hydrolysis, and bacterial fermentation. Mar. Drugs.

[10] Falch E., Rustad T., Aursand M. (2006). By-products from gadiform species as raw material for production of marine lipids as ingredients in food or feed. Process Biochem..

[11] Blanco M., Sotelo C.G., Chapela M.J., Pérez-Martín R. (2007). Towards sustainable and efficient use of fishery resources: Present and future trends. Trends Food Sci. Technol..

[12] Takahashi K., Inoue Y. (2012). Marine by-product phospholipids as booster of medicinal compounds. Adv. Food Nutr. Res..

[13] Głowacz-Rozynska A., Tynek M., Malinowska-Panczyk E., Martysiak-Zurowska D., Pawłowicz R., Kołodziejsk I. (2016). Comparison of oil yield and quality obtained by different extraction procedures from salmon (*Salmo salar*) processing byproducts. Eur. J. Lipid Sci. Technol..

[14] Pudtikajorn K., Benjakul S. (2020). Simple wet rendering method for extraction of prime quality oil from skipjack tuna eyeballs. Eur. J. Lipid Sci. Technol..

[15] Rodríguez A., Trigo M., Aubourg S.P., Medina I. (2021). Optimisation of healthy-lipid content and oxidative stability during oil extraction from squid (*Illex argentinus*) viscera by green processing. Mar. Drugs.

[16] Rubio-Rodríguez N., de Diego S.M., Beltrán S., Jaime I., Sanz M.T., Rovira J. (2012). Supercritical fluid extraction of fish oil from fish by-products: A comparison with other extraction methods. J. Food Eng..

[17] Pando M.E., Rodríguez A., Galdames A., Berríos M.M., Rivera M., Romero N., Valenzuela M.A., Ortiz J.A., Aubourg S.P. (2018). Maximization of the docosahexaenoic and eicosapentaenoic acids content in concentrates obtained from a by-product of rainbow trout (*Oncorhynchus mykiss*) processing. Eur. Food Res. Technol..

[18] Gbogouri G., Linder M., Fanni J., Parmentier M. (2006). Analysis of lipids extracted from salmon (*Salmo salar*) heads by commercial proteolytic enzymes. Eur. J. Lipid Sci. Technol..

[19] Rustad T., Storro I., Slizyte R. (2011). Possibilities for the utilisation of marine by-products. Int. J. Food Sci. Technol..

[20] Pateiro M., Gómez-Salazar J.A., Jaime-Patlán M., Sosa-Morales M.E., Lorenzo J.M. (2021). Plant extracts obtained with green solvents as natural antioxidants in fresh meat products. Antioxidants.

[21] Silva Pinho L., Palazzolli da Silva M., Thomazini M., Cooperstone J.L., Campanella O.H., da Costa Rodrigues C.E., Favaro-Trindade C.S. (2021). Guaraná (*Paullinia cupana*) by-product as a source of bioactive compounds and as a natural antioxidant for food applications. J. Food Proc. Preserv..

[22] Gil-Martín E., Forbes-Hernández T., Romero A., Cianciosi D., Giampieri F., Battino M. (2022). Influence of the extraction method on the recovery of bioactive phenolic compounds from food industry by-products. Food Chem..

[23] Gigliotti J.C., Davenport M.P., Beamer S.K., Tou J.C., Jaczynski J. (2011). Extraction and characterisation of lipids from Antarctic krill (*Euphausia superba*). Food Chem..

[24] Li C.J., Xin M.R., Sun Z.L. (2021). Selection of extraction solvents for edible oils from microalgae and improvement of the oxidative stability. J. Biosci. Bioeng..

[25] FAO (2021). Fishery Division. Species Fact Sheets. Loligo gahi (Orbigny, 1835).

[26] Bligh E., Dyer W. (1959). A rapid method of total extraction and purification. Can. J. Biochem. Physiol..

[27] Chakraborty K., Joseph D. (2015). Cooking and pressing is an effective and eco-friendly technique for obtaining high quality oil from *Sardinella longiceps*. Eur. J. Lipid Sci. Technol..

[28] AOAC (1990). Official Methods for Analysis of the Association of Analytical Chemistry.

[29] Herbes S.E., Allen C.P. (1983). Lipid quantification of freshwater invertebrates: Method modification for microquantitation. Can. J. Fish. Aquat. Sci..

[30] Raheja R., Kaur C., Singh A., Bhatia A. (1973). New colorimetric method for the quantitative determination of phospholipids without acid digestion. J. Lipid Res..

[31] Lowry R., Tinsley I. (1976). Rapid colorimetric determination of free fatty acids. J. Am. Oil Chem. Soc..

[32] Huang T., Chen C., Wefler V., Raftery A. (1961). A stable reagent for the Liebermann-Buchardt reaction. Anal. Chem..

[33] Álvarez V., Medina I., Prego R., Aubourg S.P. (2009). Lipid and mineral distribution in different zones of farmed and wild blackspot seabream (*Pagellus bogaraveo*). Eur. J. Lipid Sci. Technol..

[34] Vioque E., Holman R. (1962). Quantitative estimation of esters by thin-layer chromatography. J. Am. Oil Chem. Soc..

[35] Aubourg S.P., Trigo M., Prego R., Cobelo-García A., Medina I. (2021). Nutritional and healthy value of chemical constituents obtained from Patagonian squid (*Doryteuthis gahi*) by-products captured at different seasons. Foods.

[36] Kacem M., Sellami M., Kammoun W., Frikh F., Miled N., Rebah F.B. (2011). Seasonal variations in proximate and fatty acid composition of viscera of *Sardinella aurita*, *Sarpa salpa*, and *Sepia officinalis* from Tunisia. J. Aquat. Food Prod. Technol..

[37] Singh A., Benjakul S., Kishimura H. (2017). Characteristics and functional properties of ovary from squid *Loligo formosana*. J. Aquat. Food Prod. Technol..

[38] Toyes-Vargas E., Robles-Romo A., Méndez L., Palacios E., Civera R. (2016). Changes in fatty acids, sterols, pigments, lipid classes, and heavy metals of cooked or dried meals, compared to fresh marine by-products. Anim. Feed Sci. Technol..

[39] Saito H., Sakai M., Wakabayashi T. (2014). Characteristics of the lipid and fatty acid compositions of the Humboldt squid, *Dosidicus gigas*: The trophic relationship between the squid and its prey. Eur. J. Lipid Sci. Technol..

[40] Ortiz J., Palma Ó., González N., Aubourg S.P. (2008). Lipid damage in farmed rainbow trout (*Oncorhynchus mikiss*) after slaughtering and chilling storage. Eur. J. Lipid Sci. Technol..

[41] Barbosa R.G., Trigo M., Prego R., Fett R., Aubourg S.P. (2018). The chemical composition of different edible locations (central and edge muscles) of flat fish (*Lepidorhombus whiffiagonis*). Int. J. Food Sci. Technol..

[42] Küllenberg D., Taylor L.A., Schneider M., Massing U. (2012). Health effects of dietary phospholipids. Lipids Health Dis..

[43] Li J., Wang X., Zhang T., Huang Z., Luo X., Deng Y. (2015). A review on phospholipids and their main applications in drug delivery systems. Asian J. Pharm. Sci..

[44] Piclet G. (1987). Le poisson aliment. Composition-Intérêt nutritionnel. Cah. Nutr. Diét..

[45] Testi S., Bonaldo A., Gatta P., Badiani A. (2006). Nutritional traits of dorsal and ventral fillets from three farmed fish species. Food Chem..

[46] Sieiro M.P., Aubourg S.P., Rocha F. (2006). Seasonal study of the lipid composition in different tissues of the common octopus (*Octopus vulgaris*). Eur. J. Lipid Sci. Technol..

[47] Vairamani S., Sofia V., Sudharsan S., Vasanthkumar S., Ramasubrmanian V., Madeswaran P., Srinivasan A., Shanmugam A. (2013). Reclamation of *Loligo duvauceli* (Orbigny, 1848) digestive gland (liver) waster for the extraction of oil and its lipid composition. J. Biol. Sci..

[48] Swanson S., Block R., Mousa S. (2012). Omega-3 fatty acids EPA and DHA: Health benefits throughout life. Adv. Nutr..

[49] Ofosu F.K., Daliri E.B.M., Lee B.H., Yu X. (2017). Current trends and future perspectives on omega-3 fatty acids. Res. J. Biol..

[50] Šimat V., Vlahović J., Soldo B., Mekinić I.G., Čagalj M., Hamed I., Skroza D. (2020). Production and characterization of crude oils from seafood processing by-products. Food Biosci..

[51] Uauy R., Valenzuela A. (2000). Marine oils: The health benefits of n-3 fatty acids. Nutrition.

[52] Komprda T. (2012). Eicosapentaenoic and docosahexaenoic acids as inflammation-modulating and lipid homeostasis influencing nutraceuticals: A review. J. Funct. Foods.

[53] Kumari P., Kumar M., Reddy C.R., Jha B., Domínguez H. (2013). Algal lipids, fatty acids and sterols. Functional Ingredients from Algae for Foods and Nutraceuticals.

[54] Simopoulos A.P. (2002). The importance of the ratio of omega-6/omega-3 essential fatty acids. Biomed. Pharmacother..

